# Nonlinear dynamics in Divisia monetary aggregates: an application of recurrence quantification analysis

**DOI:** 10.1186/s40854-022-00419-5

**Published:** 2023-01-10

**Authors:** Ioannis Andreadis, Athanasios D. Fragkou, Theodoros E. Karakasidis, Apostolos Serletis

**Affiliations:** 1 International School of The Hague, Wijndaelerduin 1, 2554 BX The Hague, The Netherlands; 2 Department of Civil Engineering, University of Thessaly, 38334 Volos, Greece; 3 Condensed Matter Physics Laboratory, Department of Physics, University of Thessaly, 35100 Lamia, Greece; 4 University of Calgary, Calgary, AB Canada

**Keywords:** Divisia monetary aggregates, Recurrence plots, Moving windows, Deterministic dynamics, Stochastic structures

## Abstract

We construct recurrence plots (RPs) and conduct recurrence quantification analysis (RQA) to investigate the dynamic properties of the new Center for Financial Stability (CFS) Divisia monetary aggregates for the United States. In this study, we use the latest vintage of Divisia aggregates, maintained within CFS. We use monthly data, from January 1967 to December 2020, which is a sample period that includes the extreme economic events of the 2007–2009 global financial crisis. We then make comparisons between narrow and broad Divisia money measures and find evidence of a nonlinear but reserved possible chaotic explanation of their origin. The application of RPs to broad Divisia monetary aggregates encompasses an additional drift structure around the global financial crisis in 2008. Applying the moving window RQA to the growth rates of narrow and broad Divisia monetary aggregates, we identify periods of changes in data-generating processes and associate such changes to monetary policy regimes and financial innovations that occurred during those times.

## Introduction

In this study, we have used recurrence plots (RPs) and performed recurrence quantification analysis (RQA) to investigate the dynamics of the United States Divisia monetary aggregates, initially developed by Barnett (1980) and currently maintained at the Center for Financial Stability (CFS) in New York City. In this regard, Barnett and Chen (1988) claimed a successful chaos detection in Divisia monetary aggregates. Their conclusion was further confirmed by DeCoster and Mitchell (1991, 1994). This published claim of successful chaos detection has generated considerable controversies, as in Ramsey and Rothman (1994) and Ramsey et al. (1988). It has also motivated further investigations, as in Barnett et al. (1995, 1997), Serletis (1995), and Serletis and Andreadis (2000). In the study conducted by Barnett and Serletis (2000), they provided extensive discussions of controversies that have arisen as regards available tests and results (see also Serletis and Shintani 2006).

In this study, we aim to construct RPs and conduct RQA to investigate the dynamics of Divisia monetary aggregates. In recent years, there have been many applications of RPs, introduced by Eckmann et al. (1987), and RQA, introduced by Zbilut and Webber (1992) and Marwan (2003), in the analysis of economic and financial time series (e.g., Strozzi et al. 2002; Fabretti and Ausloos 2005; Barkoulas 2008; Faggini 2013, 2014; and Han 2019). Moreover, RPs have been used in the analysis of nonstationary time series (e.g., Facchini et al. 2005).

As Fabretti and Ausloos (2005, p. 671) put it, “Recurrence Plot (RP) and Recurrence Quantification Analysis (RQA) are signal numerical analysis methodologies able to work with nonlinear dynamical systems and nonstationarity.” RPs are known as graphical tools based on phase space reconstruction (Eckmann et al. 1987), and RQA is a statistical quantification of RPs (Zbilut and Webber 1992). These methodologies do not intend to provide chaos evidence and are used with nonstationary and noisy time series to detect phase transitions and rare events.

We use the latest vintage of Divisia aggregates, maintained within the CFS program Advances in Monetary and Financial Measurement called CFS Divisia aggregates and documented in detail in Barnett et al. (2013). We use monthly data, from January 1967 to December 2020, which is a sample period that includes the extreme economic events of the 2007–2009 global financial crisis and part of the coronavirus disease 2019 (COVID-19) pandemic. We make comparisons between narrow Divisia money measures (those at the M1, M2, M2M, MZM monetary aggregation levels) and broad Divisia money measures (those at M3, M4-, M4 monetary aggregation levels).

Barnett (1978, 1980) developed Divisia monetary aggregates. In contrast to the simple sum monetary aggregates constructed by most central banks, Divisia monetary aggregates are found to be consistent with economic aggregation theory. Specifically, simple sum monetary aggregates are consistent with economic aggregation theory only if liquid assets are perfect substitutes with the same user cost. However, monetary assets yield interest, whereas currency does not; thus, the assumption that simple sum monetary aggregates are based on is deemed unreasonable. Divisia monetary aggregates do not assume perfect substitutions among component assets and hence permit different user costs of component assets. Barnett (1978, 1980) also demonstrated that Divisia aggregates represent superior measurements of liquidity services as compared with simple sum monetary aggregates. As a result, all modern formal investigations of the impact of money on economic activities are performed using Divisia aggregates.

Our results are consistent with Barkoulas (2008) and supportive of a high-order deterministic structure for each CFS Divisia monetary aggregate. We have also utilized the moving window and epoch methodologies of Trulla et al. (1996) and Bastos and Caiado (2011) to the growth rates of Divisia monetary aggregates and identified the effects of changes in monetary policy strategies and various innovations in the US financial service landscape on the dynamics of Divisia monetary aggregates.

This paper is organized as follows: The “Divisia monetary aggregates” section briefly provides the theoretical foundations of Divisia monetary aggregates. The “Data” section discusses CFS data and provides graphical representations of narrow and broad Divisia monetary aggregates, in logarithms and in growth rates. In “RPs” and “RQA” sections we present the RPs and RQA of Divisia aggregates. In “Epochs in the growth rates of Divisia monetary aggregates” section we present the application of the moving window method to the growth rates of narrow and broad Divisia monetary aggregates. In “Conclusion” section provides the conclusion.

## Divisia monetary aggregates

Unlike the simple sum monetary aggregates currently used by most central banks worldwide, Divisia monetary aggregates, invented by Barnett (1978, 1980), do not assume perfect substitutability among their component assets and allow for different user costs of their components. The formula for the user cost (in real terms) of asset *i*, denoted by *π*_*it*_, was derived by Barnett (1978) and is given as follows:

$$\pi_{it} = \frac{{R_{t} - r_{it} }}{{1 + R_{t} }},$$

where ${R}_{t}$ is the rate of return on the benchmark asset and ${r}_{it}$ is the rate of return on asset *i*. The user cost can be interpreted as the interest foregone by holding a dollar’s worth of liquid asset *i*.

With data on user costs and quantities of component assets, one can calculate the expenditure share of asset *i* as follows:

$$s_{it} = \frac{{\pi_{it} m_{it} }}{{\mathop \sum \nolimits_{i = 1}^{n} \pi_{it} m_{it} }},$$

where *m*_*it*_ is the balance (in real terms) of asset *i* in period *t*. Then, the (discrete time) growth rate of a Divisia aggregate is given by the weighted average of its component growth rate, with the weight being the expenditure share of the respective component, as follows:

$$dlogM_{t} = \mathop \sum \limits_{i = 1}^{n} s_{it} dlogm_{it} .$$

Divisia share weights are deemed significant. They depend on all prices and quantities and weight component growth rates to give the growth rates of Divisia aggregates.

Over the years, Barnett (1978, 1980), Belongia (1996), Hendrickson (2014), Serletis and Gogas (2014), Belongia and Ireland (2014, 2015, 2016), Ellington (2018), Dai and Serletis (2019), Serletis and Xu (2020, 2021), and Xu and Serletis (2022) have demonstrated the superiority of Divisia monetary aggregates over simple sum aggregates. In fact, these authors revealed that Divisia aggregates are superior to the simple sum aggregates currently used by central banks. Moreover, Barnett (2016), Jadidzadeh and Serletis (2019), and Dery and Serletis (2021) argued that we should be using broad monetary aggregates, as opposed to the narrow ones.

In this study, we provide further evidence in support of broad Divisia monetary aggregates by applying moving window and epoch theories (see Trulla et al. 1996 and Bastos and Caiado 2011) to the growth rates of the aggregates.

## Data

We use monthly United States data, from January 1967 to December 2020. This sample period includes the global financial crisis and part of the coronavirus recession. It is also dictated by the availability of Divisia monetary aggregates. The aggregates are maintained within CFS. In this research, we have compared narrow Divisia aggregates (at M1, M2, M2M, MZM aggregation levels) and broad Divisia aggregates (at M3, M4-, and M4 levels). For a detailed discussion of the data and methodology for the calculation of Divisia aggregates, see Barnett et al. (2013) and http://www.centerforfinancialstability.org.

**Fig. 1:**
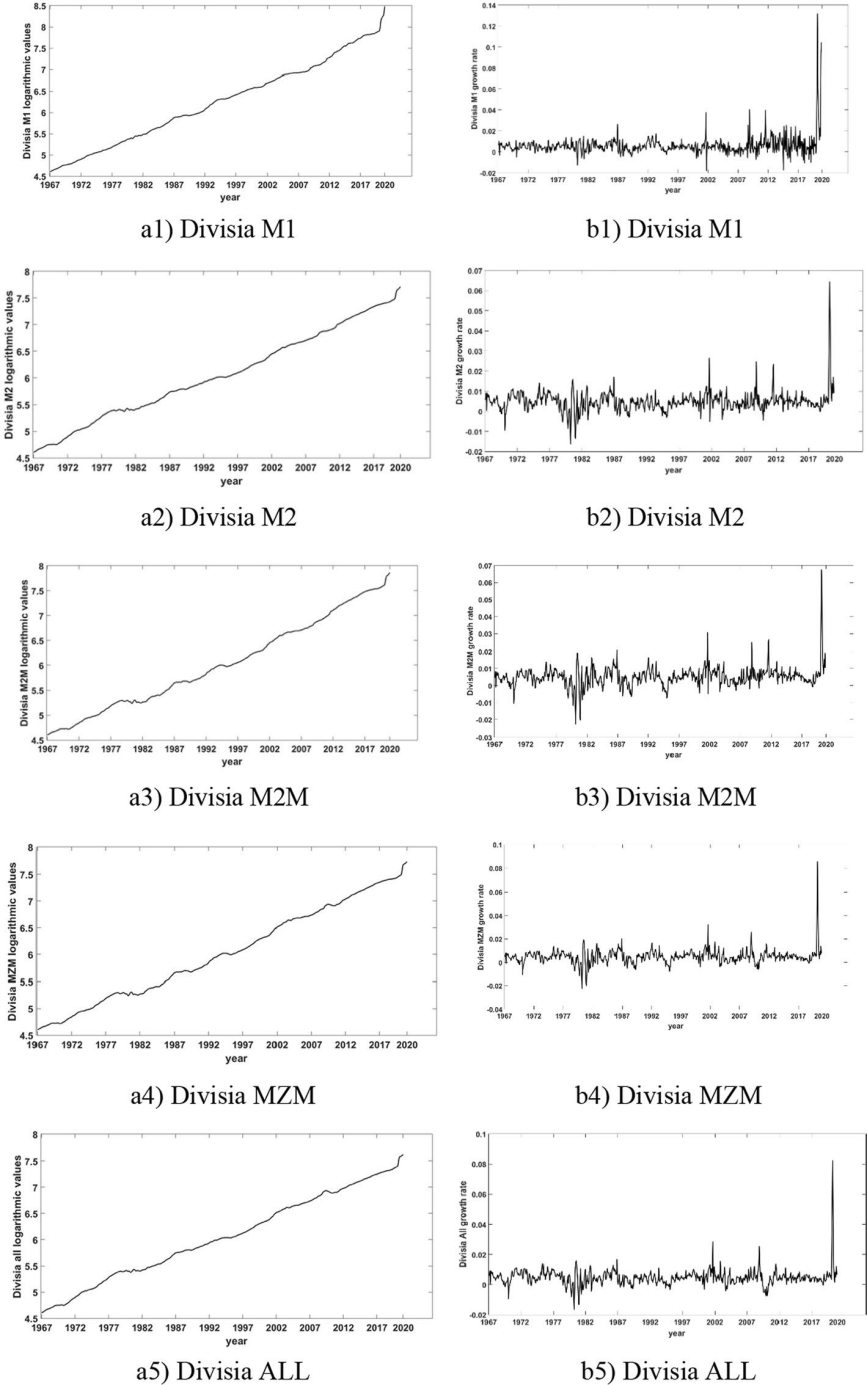
Logarithmic values (in Panel **a**) and growth rates (in Panel **b**) of narrow Divisia monetary aggregates

**Fig. 2:**
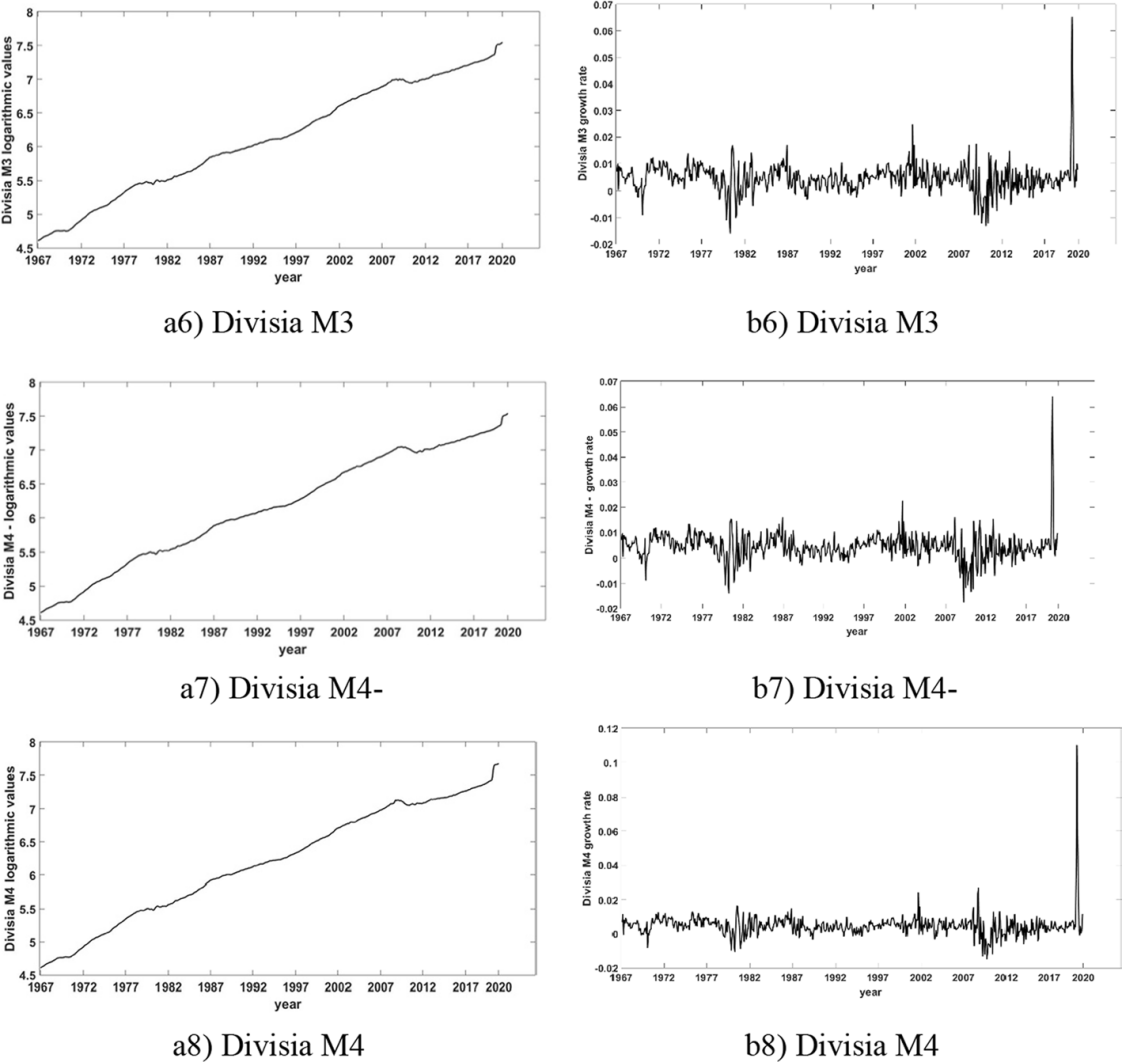
Logarithmic values (in Panel **a**) and growth rates (in Panel **b**) of broad Divisia aggregates

In Figs. 1 and 2, we present the logged levels (in Panel (a)) and the growth rates (in Panel (b)) of all aggregates. We observe that they all trend steadily upwards but also follow slightly distinct paths. The general pattern in all graphs is the persistent upward trend in logarithmic levels and the significant variability in growth rates (changes in logarithmic levels); see Dery and Serletis (2021) for detailed discussions of these differences.

## RPs

RPs were first introduced by Eckmann et al. (1987) to extract the qualitative characteristics of a dynamical system. They are described to be a graphical tool associated with the trajectory of the phase space of an underlying system. Since their introduction, RPs have been utilized in various areas (for a review, see Marwan et al. 2008). The purpose of RPs is to extract qualitative characteristics of a dynamical system based on the texture appearing on them and locate transitions of its dynamical behavior. Based on the texture, one can identify random-like, periodic, and chaotic behaviors. The RPs of random systems show a cloud of points, which are opposite to the RPs of systems that emanate from deterministic, either chaotic or nonchaotic behaviors where various patterns appear (for detailed descriptions of these patterns and their significance, we refer to Marwan et al. 2015). Moreover, a set of tools have been developed to quantify the above behaviors of RPs, such as DET, RR, and TT indexes, which are described in the above reference and briefly below in the section corresponding to RPs. Changes in visual structures and modifications in measured quantities can help effectively and objectively identify transitions in system behaviors.

Initially, we recall the method of creating a recurrence plot (see Eckmann et al. 1987 and Marwan 2006 for further details). Consider time series $T$ with K points, denoted as $T = \left\{ {T_{1} , T_{2} , \ldots , T_{K} } \right\}$, with $T_{i} \in {\mathbb{R}}$, and $1 \le i \le K$. We define an embedding of time series *T* (see Packard et al. (1980) and Takens (1981)) by fixing embedding dimension $d_{E,T}$ and delay time $\tau_{T}$. We define the reconstructed time series $S$ that possesses $L_{T}$ points, $L_{T} = K - \left( {d_{E,T} - 1} \right)\tau_{T} ,$ as $S = \left\{ {S_{1} , S_{2} , \ldots ,S_{i} , \ldots ,S_{{L_{T} }} } \right\}$, with $S_{i} = \left( {T_{i} , T_{{i + \tau_{T} }} , \ldots , T_{{i + \left( {d_{E,T} - 1} \right)\tau_{T} }} } \right) \in R_{{}}^{{d_{E,T} }} , 1 \le i \le L_{T}$. Then, we choose threshold value *r*, which is also well known as a cut-off distance. Next, we consider the recurrence plot Lattice $L_{T} \times L_{T}$ with points $\left( {i,j} \right), 1 \le i,j \le L_{T}$. Finally, for each value of $\left( {i,j} \right)$ of the recurrence plot Lattice and a positive threshold value $r$, we define function $f: L \times L \to \left\{ {1, 0} \right\}$ as follows:

$$f\left( {i,j} \right) = \begin{array}{*{20}l} {1, S_{j} \in B\left( {S_{i} ,r} \right) } \hfill & {i,j recurrent} \hfill \\ {0, S_{j} \notin B\left( {S_{i} ,r} \right),} \hfill & {otherwise} \hfill \\ \end{array}$$

where *B* $\left( {S_{i} ,r} \right)$ denotes the neighborhood with center $S_{i}$ and threshold $r$; it consists of all the points that are within distance *r* from center *S*_i_.

In the recurrence plot Lattice, we plot with black the recurrent points and with white the nonrecurrent points. The distribution of these black and white points in RPs creates various patterns called textures that reflect various dynamical system properties (see Eckmann et al. 1987). In this work, we use the MATLAB (MATLAB, 2008) software to plot the RPs.

In Table 1, we present the values for embedding dimension, delay time, and threshold that are used to construct RPs for narrow aggregates (Divisia M1, Divisia M2M, Divisia MZM, Divisia M2, Divisia ALL) and of their growth rates. To estimate embedding dimension, we apply the false nearest neighbor method using a criterion of below 10% falseness of nearest neighbors (see Kennel et al. 1992). For the calculation of the optimal delay time, we use the first minimum of the average mutual information as in Fraser and Swinney (1986). Finally, as regards the recurrence plot threshold, we select a recurrence rate of 3% when we use growth rates and 2% when we use logged aggregate levels, ensuring the recurrence matrix to be sparse enough to succeed in retaining system information, as suggested by Zbilut et al. (1992). The selection of these values is done by fixing the recurrence rate at 3%. Table 2 provides similar information for broad monetary aggregates (Divisia M3, Divisia M4-, and Divisia M4).

**Table 1:** Parameter values for the constructions of RPs of narrow aggregates

Parameter	M1	M2	M2M	MZM	ALL
*(a) Logged Series*					
Delay time	25	23	24	25	24
Embedding dimension	2	2	2	2	2
Threshold	0.075	0.063	0.067	0.068	0.062
*(b) Growth rate Series*					
Delay time	2	2	3	2	3
Embedding dimension	5	5	5	6	4
Threshold	0.0055	0.004	0.0049	0.0054	0.0033

**Table 2:** Parameter values for the constructions of RPs of broad aggregates

Parameter	Divisia M3	Divisia M4-	Divisia M4
*(a) Logged Series*			
Delay time	24	24	24
Embedding dimension	2	2	2
Threshold	0.06	0.059	0.061
*(b) Growth rate Series*			
Delay time	2	2	2
Embedding dimension	5	5	6
Threshold	0.0045	0.0045	0.0054

**Fig. 3:**
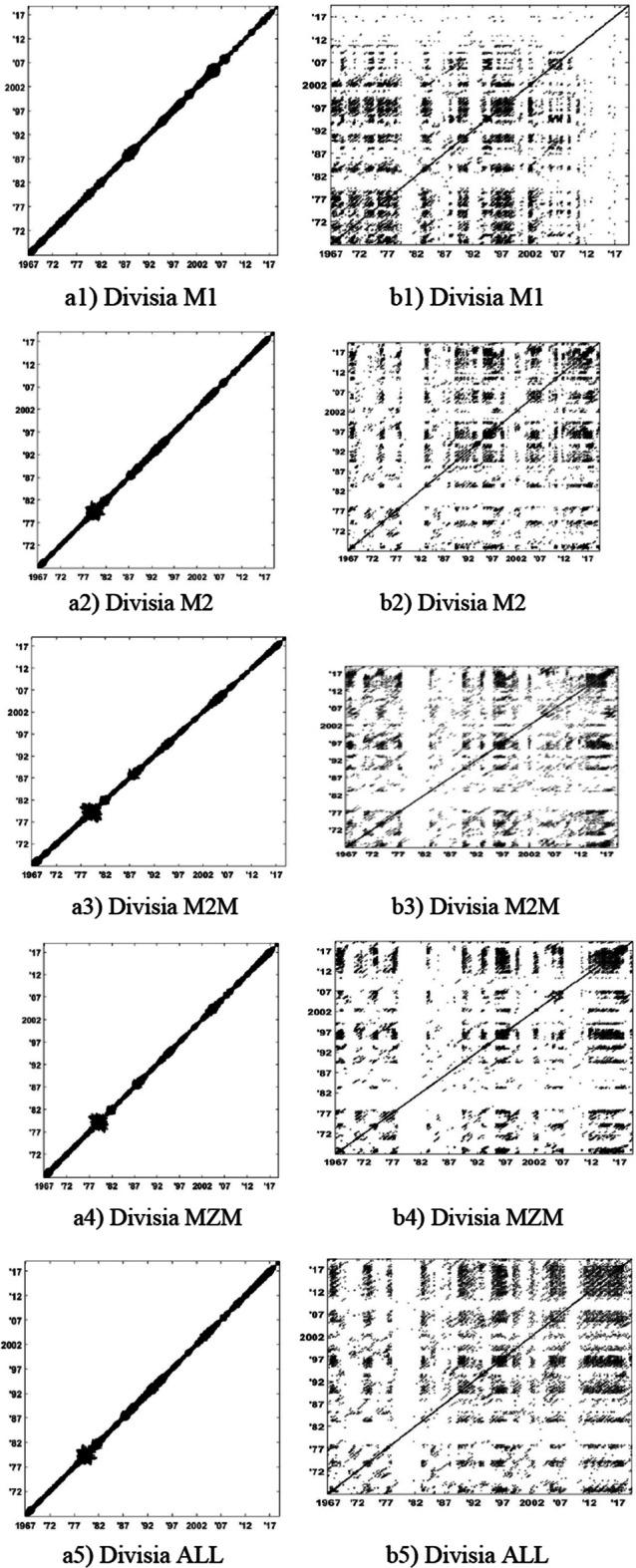
RPs of narrow Divisia monetary aggregates: Logarithmic values in Panel (**a**) and growth rates in Panel (**b**)

**Fig. 4:**
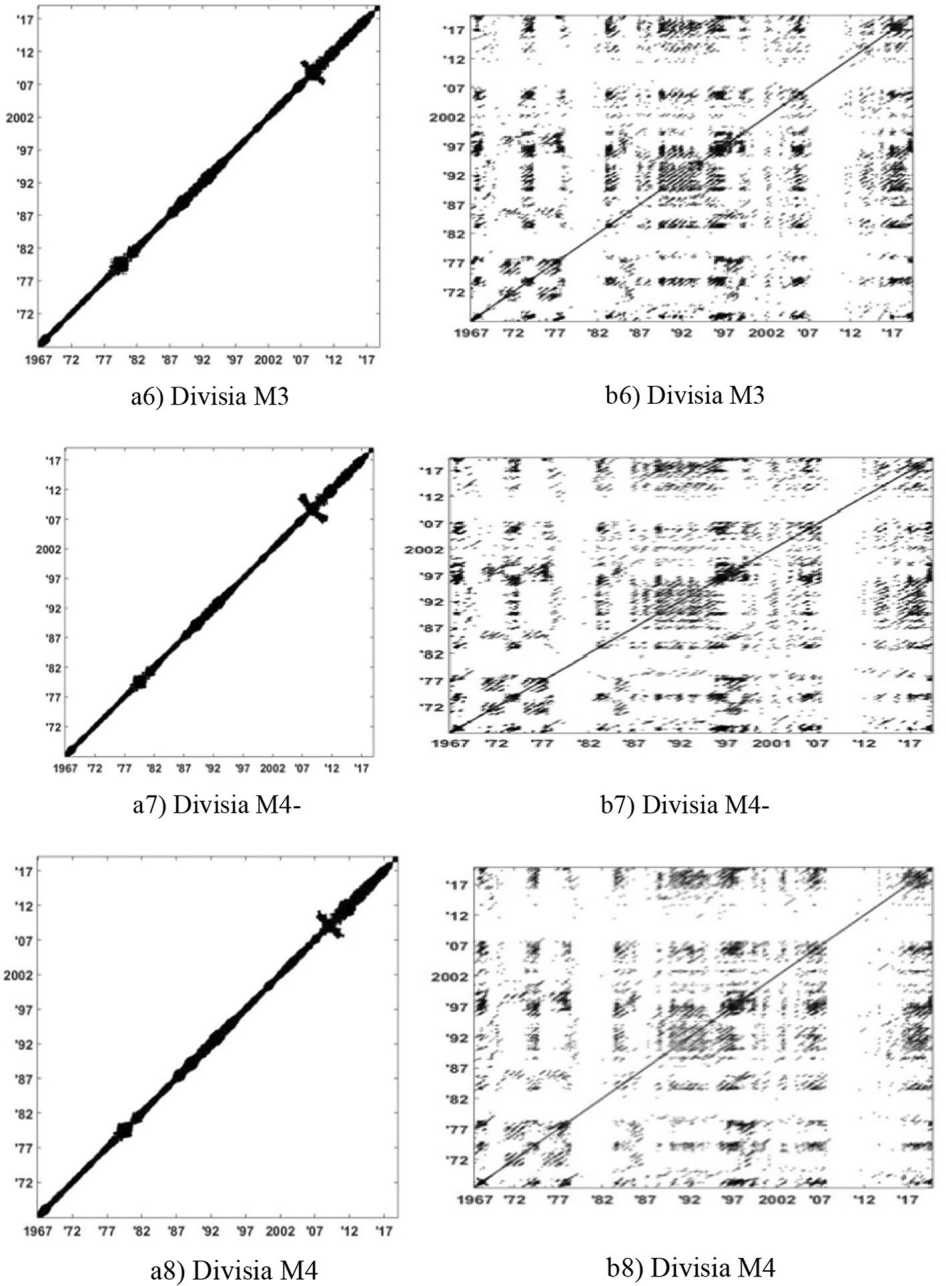
RPs of broad Divisia monetary aggregates: Logarithmic values in Panel (**a**) and growth rates in Panel (**b**)

The RPs of narrow Divisia monetary aggregates are illustrated in Fig. 3, and those of broad Divisia monetary aggregates are shown in Fig. 4. We show the RPs of the logged series in Panel (a) and of their growth rate series in Panel (b).

As can be seen from the RPs in Panel (a) of Figs. 3 and 4, for narrow and broad Divisia aggregates, the recurrent points are concentrated around the main diagonal with high density. This observation is consistent with the logged series that present a constant trend (mainly increasing) because they are monthly and do not present particular variations. Thus, values quite close seem to be related; thus, points (i, j) and (i + 1, j + 1) on RPs seem to be related with a kind of law. This case is valid over a short time frame because after some steps (several months), values vary in important ways and seem unrelated. However, RPs using growth rates in Panel (b) of Figs. 3 and 4 indicate different behaviors. We observe in many cases some short diagonal lines, which indicate deterministic behaviors because successive points in the phase space are linked to one another. These regions are separated by white regions of variable lengths indicating considerable abrupt system perturbations due to external reasons, as can be seen in several periods and may be attributed to significant financial events of short or long durations.

We have also observed that the RPs of Divisia time series contain large white areas starting from the upper left and lower right corners to the main diagonal (drift). These recurrence plot textures appear due to continuous slowly increasing values with almost no fluctuations. Thus, a drift depicts slowly varying system parameters.

Finally, an interesting observation is that the RPs of broad Divisia monetary aggregates had an additional drift structure around 2008, which is the time of the global financial crisis.

## RQA

ZBILUT and Webber (1992) proposed various statistical measures to quantify pattern presence in RPs. The statistical quantification of RPs is known as RQA. It provides a good insight on the way that recurrence points are distributed in RPs. RQA measures are used as deterministic or chaotic behavior indicators. These measures are defined in Table 3 (see Fabretti and Ausloos 2005 and Bastos and Caiado 2011 for further details).

**Table 3:** RQA measures

Definition	Notation	Explanation
Recurrence Rate (RR) or %recurrence	$RR = \frac{1}{{N^{2} }}\mathop \sum \limits_{i,j = 1}^{N} R_{i,j}$ $$R_{i,j} = \left\{ {\begin{array}{*{20}c} {1, \left( {i,j} \right) recurrent} \\ {0, otherwise} \\ \end{array} } \right.$$	High Recurrence Rate values indicate recurrent states that could be due to a deterministic behavior while chaotic states are associated with low values of the Recurrence Rate
Determinism (DET)	$DET = \frac{{\mathop \sum \nolimits_{{l = l_{min} }}^{N} l \cdot Pd\left( l \right)}}{{\mathop \sum \nolimits_{i,j}^{N} R_{i,j} }}$ where *Pd(l)* is the histogram of the lengths *l* of the diagonal lines	The presence of diagonal lines indicates the existence of a deterministic structure. DET reveals also information relevant to the duration of a stable interaction
Average Length (L)	$L = \frac{{\mathop \sum \nolimits_{{l = l_{min} }}^{N} l \cdot Pd\left( l \right)}}{{\mathop \sum \nolimits_{{l = l_{min} }}^{N} Pd\left( l \right)}}$	This measure refers to the diagonal line length. Small L values reveal processes with stochastic or chaotic behavior while big values indicate a deterministic process. The L value is related to the DET value, as both count the number of recurrent points on the diagonal structures
Laminarity (LAM)	$LAM = \frac{{\mathop \sum \nolimits_{{l = l_{min} }}^{N} l \cdot Pv\left( l \right)}}{{\mathop \sum \nolimits_{l = 1}^{N} lPv\left( l \right)}}$ where *Pv(l)* is the histogram of the lengths of the vertical lines included in the recurrence plot	Laminarity is a measure of the appearance of laminar states indicative of intermittency. The lower the LAM value, the more stable the system is
Trapping Time (TT)	$TT = \frac{{\mathop \sum \nolimits_{{l = l_{min} }}^{N} lPv\left( l \right)}}{{\mathop \sum \nolimits_{{l = l_{min} }}^{N} Pv\left( l \right)}}$ Wh where *Pv(l)* is the histogram of the lengths of the vertical lines	The Trapping Time measure indicates the slowing variation of the values of the time series through time. High TT values indicate a non-fluctuating, slow changing series

In Tables 4 and 5, we present the RQA for logged Divisia monetary aggregates and their growth rates, respectively. According to the RQA (using a recurrence rate of around 2%), the values of measures in Table 4 for the logged series are noted to be high; dense structures form dense horizontal and diagonal lines. However, the values of the same measures for the growth rates of aggregates in Table 5 are low, which indicates a process in which periodicities do not dominate; sharp fluctuations and possible changes occur in the series dynamics.

**Table 4:** RQA of Divisia aggregates (in log levels)

Aggregate	RR	DET	LAM	L	TT
*Narrow monetary aggregates*					
Divisia M1	0*.*0309	0*.*9800	0*.*9974	30*.*13	18*.*92
Divisia M2	0*.*0308	0*.*9928	0*.*9986	32*.*42	18*.*07
Divisia M2M	0*.*0302	0*.*9951	0*.*9987	27*.*85	17*.*37
Divisia MZM	0*.*0308	0*.*9923	0*.*9987	28*.*76	18*.*02
Divisia All	0*.*0307	0*.*9910	0*.*9977	25*.*49	17*.*05
*Broad monetary aggregates*					
Divisia M3	0*.*0306	0*.*9914	0*.*9972	26*.*98	17*.*27
Divisia M4-	0*.*0307	0*.*9910	0*.*9971	25*.*49	17*.*05
Divisia M4	0*.*0301	0*.*9910	0*.*9974	27*.*17	17*.*29

**Table 5:** RQA of Divisia aggregates (in growth rates)

Aggregate	RR	DET	LAM	L	TT
*Narrow monetary aggregates*					
Divisia M1	0.03171	0.1858	0.3763	3.437	2.798
Divisia M2	0.0323	0.1897	0.3751	3.249	2.891
Divisia M2M	0.0316	0.2648	0.4284	2.352	2.707
Divisia MZM	0.0306	0.1894	0.4222	4.027	3.120
Divisia All	0.0319	0.1739	0.3373	2.165	2.357
*Broad monetary aggregates*					
Divisia M3	0.0323	0.2580	0.4494	3.832	3.027
Divisia M4-	0.0307	0.2335	0.4160	3.860	2.919
Divisia M4	0.0307	0.2305	0.4239	3.837	2.726

The high laminarity values for broad Divisia monetary aggregates can be considered strong indications of nonlinear deterministic behaviors. The RR values for logged Divisia aggregates (in Table 4) and their growth rates (in Table 5) are nearly equal. The LAM values are higher than the DET values for the logged Divisia series, which presents trend-like behaviors with continued increases and nearly constant regions in contrast to their growth rates, which then present significant variations, especially in several regions.

As the Divisia M4 monetary aggregate has attracted attention in recent empirical investigations (e.g., Jadidzadeh and Serletis 2019 and Dery and Serletis 2021), we elaborate briefly below a detailed analysis of the Divisia M4 monetary aggregate based on the results in Tables 4 and 5, keeping in mind that our results do not favor the Divisia M4 aggregate over the other broad Divisia aggregates. In the case of the logged Divisia M4 monetary aggregate, we observe a high DET value (0.9910) and an average line length L (27.17), referring to diagonal lines, and a high LAM value (0.9974) and a trapping time TT value (17.29), referring to vertical lines. These values depict a strong deterministic process, that is, the values of the logged Divisia M4 monetary aggregate present periodicities during time evolution. Moreover, high TT values reveal states that are trapped in time, suggesting that the Divisia M4 aggregate is relatively stable. Moreover, laminarity values are higher than determinism values, providing the information of a more time-trapped dynamic process.

These observations are further supported with the similar behavior of the Divisia M4 growth rate series with a LAM value, which is higher than its DET value. In addition, a high TT value for the Divisia M4 growth rate series further supports the stability of the Divisia M4 monetary aggregate.

In what follows, we investigate the deterministic structures of narrow and broad Divisia monetary aggregates by applying the moving window method (see Trulla et al. 1996).

## Epochs in the growth rates of Divisia monetary aggregates

In this section, we closely look into the RPs of the logged levels and growth rates of Divisia aggregates. As illustrated in Figs. 3 and 4, a concentration of recurring points is noted to occur around the main diagonal with various areas of different behaviors. This morphology indicates a nearly continuous and monotonic variation, which is true because we have a continuous increase in time series values, and due to this increase, various regions become uncorrelated, reflected by the large white areas for the rest of its recurrence plot. These different structures show us that the system under study is subject to phase transitions. The RPs of their growth rates present quite different behaviors as far as the structures are concerned. The structures are different with small parallel lines interrupted quite often by large white vertical regions, which are representative of the fluctuating behavior of the series; the white areas correspond to large perturbations.

**Fig. 5:**
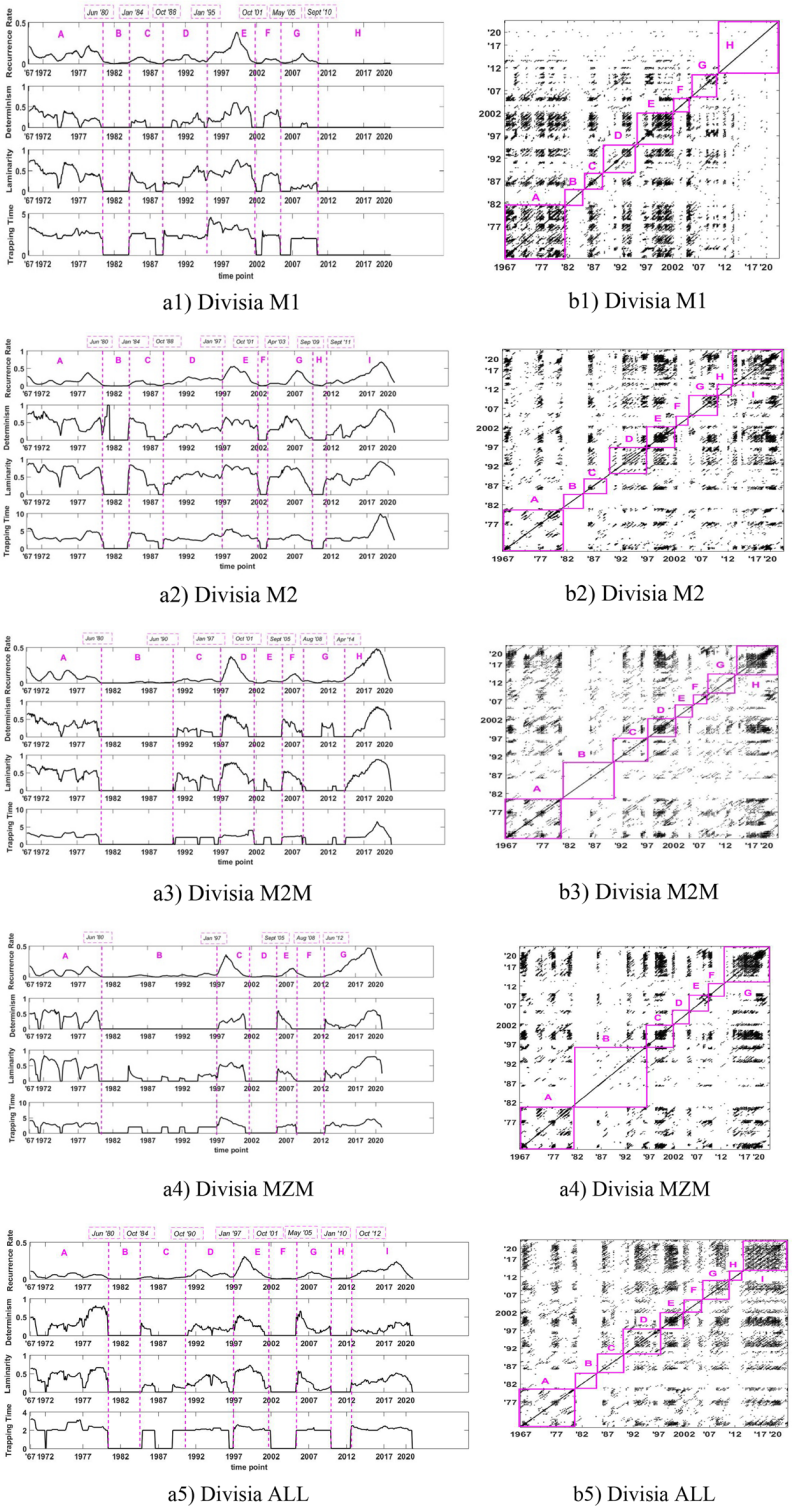
RQA of the growth rates of narrow Divisia monetary aggregates in Panel (**a**) in 611 quarterly rolling windows (the estimated dates (month/year) of phase transitions are shown on the top) and in Panel (**b**) in their RPs with regions

**Fig. 6:**
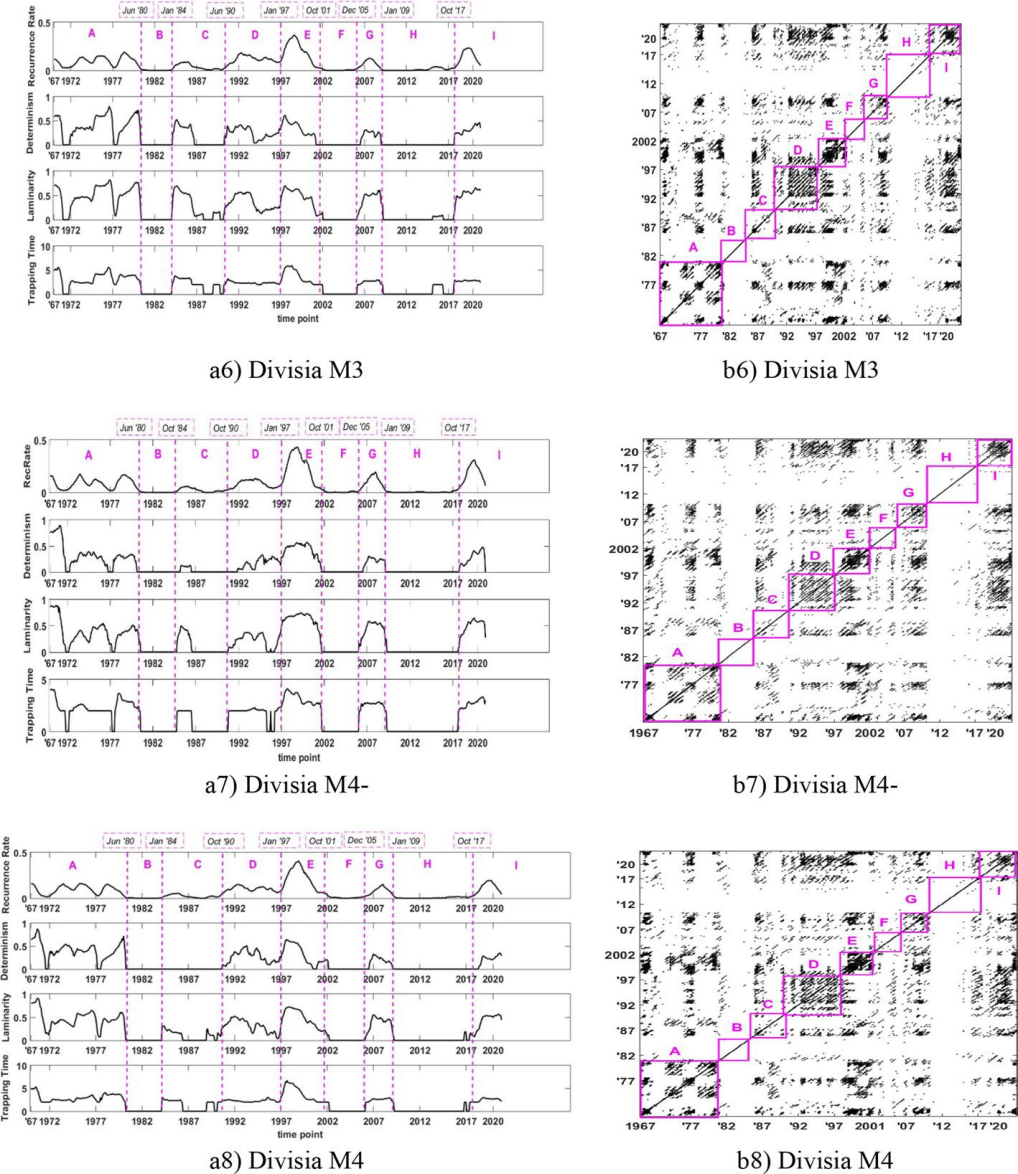
RQA of the growth rates of broad Divisia monetary aggregates in Panel (**a**) in 611 quarterly rolling windows (the estimated dates (month/year) of phase transitions are shown on the top) and in Panel (**b**) in their RPs with regions

Although visual inspection is deemed useful, we follow Marwan et al. (2007) and employ an epoch analysis, which is also known as a moving window (see Trulla et al. 1996), to localize in a detailed way the series dynamics. Epochs are equidistant periods that are calculated along the main diagonal of the recurrence plot and help us locate possible phase transitions during system evolution (see Bastos et al. 2011). We calculate the values of various RQA measures discussed in Sect. 5, over sliding windows with successive points of RPs, that is, densities of recurrent points (recurrence rates) and densities of recurrent points in diagonal structures (determinism) and in horizontal structures (laminarity and trapping time). Significant variations of these quantities from one window to another can help detect abrupt changes in system dynamics. Exploring various values of equidistant periods, we choose a moving window of 36 observations, corresponding to a 3-year period. We choose the length of 36 through visual inspections, as suggested in Facchini et al. (2007), because for small values, points in corresponding RPs are found insufficient to extract significant values for RQA measures. The corresponding results appear in Figs. 5 and 6.

We plot the growth rates of narrow (in Fig. 5) and broad (in Fig. 6) Divisia monetary aggregates. Panel (a) presents 611 quarterly rolling windows (the estimated dates (month/year) of phase transitions are shown on the top), whereas Panel (b) shows their RPs with regions. We indicate seven regions, namely, A, B, C, D, E, F, and H, in chronological order. These regions stay the same for broad Divisia monetary aggregates (see Panel (a) of Fig. 6); in contrast to the case of narrow Divisia monetary aggregates, see Panel (a) of Fig. 5.

In Table 6, we present the dates around which the growth rates of narrow and broad Divisia monetary aggregates exhibit significant dynamical changes.

**Table 6:** Dates when the dynamics of the growth rates of Divisia monetary aggregates exhibit significant dynamical changes

	Narrow monetary aggregates					Broad monetary aggregates		
Date	M1	M2	M2M	MZM	All	M3	M4-	M4
Jun. 1980	YES	YES	YES	YES	YES	YES	YES	YES
Jan. 1984	YES	YES				YES	YES	YES
Oct. 1988	YES	YES						
Jun 1990			YES			YES		
Oct. 1990					YES		YES	YES
Jan. 1997		YES	YES	YES	YES	YES	YES	YES
Oct. 2001	YES	YES	YES		YES	YES	YES	YES
Sept. 2005			YES	YES				
Dec. 2005						YES	YES	YES
Aug. 2008			YES	YES				
Jan. 2009						YES	YES	YES
Oct 2017						YES	YES	YES

As per our findings, in June 1980, January 1997, and October 2001, almost all eight Divisia monetary aggregates were subject to changes. In this regard, the time around 1980 is a transition period for the Federal Reserve monetary policy. In the 1970s, the operating target of the US monetary policy was the federal funds rate with the monetary aggregates serving as intermediate targets. However, the Fed switched to a monetary policy targeting in October 1979, using nonborrowed reserves as the primary operating instrument and monetary aggregates as intermediate targets. This policy was abandoned 3 years later, in October 1982, and the Fed returned to a smoothing interest rate policy.

Changes in January 1997 and October 2001 may be related to innovations in the financial service industry and the financial deregulation that took place around that time (e.g., Calmès and Théoret 2020a; 2020b; 2021). Specifically, in the mid-1990s, a financial innovation known as “sweep technology” enabled banks in the United States to avoid “taxes” from reserve requirements. At the end of a business day, commercial banks could sweep out of the checking account of a corporation any balances above a certain amount and invest in overnight securities in the overnight interbank market. The swept-out funds were not subject to reserve requirements because they were unclassified as checkable deposits. Sweep accounts then became popular by the end of the 1990s and early 2000s in the United States.

Broad Divisia monetary aggregates exhibit similar dynamics because their transitions take place at about the same time. By contrast, we observe different dynamics in narrow Divisia aggregates. Moreover, we find that broad Divisia aggregates exhibit dynamical changes more often than their narrow counterparts. These results are consistent with the analysis by Dery and Serletis (2021) who provided comparisons between narrow and broad Divisia monetary aggregates, using different methods from ours, in their investigation of the information content of Divisia aggregates. They used the methodology suggested by Kydland and Prescott (1990) and produced cyclical components using the Hamilton (2018) and Hodrick and Prescott (1980) filters. They found that all narrow Divisia monetary aggregates are acyclical, whereas broad Divisia monetary aggregates are weakly procyclical. Our results, however, do not speak for, or against, any of the Divisia monetary aggregates as preferred measures of money, as we only focused on dynamic aggregate behaviors.

## Conclusion

We use RPs and RQA to investigate the dynamics of Divisia monetary aggregates. Evidence indicates a nonlinear dynamical process in which periodicities do not dominate, further supporting the result of (Barkoulas 2008) on a reservation of a possible chaotic explanation of Divisia money measures.

We also apply the moving window method of Trulla et al. (1996) to the growth rates of narrow and broad Divisia monetary aggregates. Our results reveal their changes during the study period. The epochs method highlights the full dynamic behavior of the system under study because it identifies with relative precision the times when phase transitions take place. Our analysis reveals the effects of changes in monetary policy strategies and various innovations in the US financial services landscape that take place over the sample period.

Motivated by the need to solve the “Barnett critique,” we have completely ignored the simple sum aggregates provided by the Fed. We only utilized new CFS Divisia monetary aggregates and made comparisons between narrow and broad measures. In this regard, as noted by Barnett et al. (2013), the components of Divisia aggregates closely mirror their simple sum counterparts provided by the Fed. However, the Fed stopped reporting the (simple sum) M3 monetary aggregate in March 2006, and the broadest aggregate that is currently reported is (simple sum) M2, which excludes a great deal of the quantity of financial intermediation in the economy.

## Abbreviations

CFS: Center for financial stability
DET: Determinism
US: United States
L: Average length
LAM: Laminarity RPs
RPs: Recurrence plots
RQA: Recurrence quantification analysis
RR: Recurrence rate
TT: Trapping time

## References

[CR1] Barkoulas JT (2008). Testing for deterministic monetary chaos: metric and topological diagnostics. Chaos Solitons Fractals.

[CR2] Barnett WA (1978). The user cost of money. Econ Lett.

[CR3] Barnett WA (1980). Economic monetary aggregates: an application of aggregation and index number theory. J Econom.

[CR4] Barnett WA, Cord RA, Hammond J (2016). Friedman and Divisia monetary measures. Contributions to economics and public policy.

[CR5] Barnett WA, Chen P, Barnett WA, Berndt E, White E (1988). The aggregation-theoretic monetary aggregates are chaotic and have strange attractors: an econometric application of mathematical chaos. Dynamic econometric modeling.

[CR6] Barnett WA, Serletis A (2000). Martingales, nonlinearity, and chaos. J Econ Dyn Control.

[CR7] Barnett WA, Gallant AR, Hinich MJ, Jungeilges JA, Kaplan DT, Jensen MJ (1995). Robustness of nonlinearity and chaos tests to measurement error, inference method, and sample size. J Econ Behav Organ.

[CR8] Barnett WA, Gallant AR, Hinich MJ, Jungeilges JA, Kaplan DT, Jensen MJ (1997). A single-blind controlled competition among tests for nonlinearity and chaos. J Econom.

[CR9] Barnett WA, Liu J, Mattson RS, van den Noort J (2013). The new CFS Divisia monetary aggregates: design, construction, and data sources. Open Econ Rev.

[CR10] Bastos JA, Caiado J (2011). Recurrence quantification analysis of global stock markets. Phys A.

[CR11] Belongia MT (1996). Measurement matters: recent results from monetary economics reexamined. J Polit Econ.

[CR12] Belongia MT, Ireland PN (2014). The Barnett critique after three decades: a new Keynesian analysis. J Econom.

[CR13] Belongia MT, Ireland PN (2015). Interest rates and money in the measurement of monetary policy. J Bus Econ Stat.

[CR14] Belongia MT, Ireland PN (2016). Money and output: Friedman and Schwartz revisited. J Money Credit Bank.

[CR15] Calmès C, Théoret R (2020). The impact of universal banking on macroeconomic dynamics: a nonlinear local projection approach. Borsa Istanbul Rev.

[CR16] Calmès C, Théoret R (2020). Bank fee-based shocks and the U.S. business cycle. N Am J Econom Financ.

[CR17] Calmès C, Théoret R (2021). Portfolio analysis of big US banks’ perfor-mance: the fee business lines factor. J Bank Regul.

[CR18] Dai W, Serletis A (2019). On the Markov switching welfare cost of inflation. J Econ Dyn Control.

[CR19] DeCoster GP, Mitchell DW (1991). Nonlinear monetary dynamics. J Bus Econom Stat.

[CR20] DeCoster GP, Mitchell DW (1994). A reply. J Bus Econom Stat.

[CR21] Dery C, Serletis A (2021). Interest rates, money, and economic activity. Macroecon Dyn.

[CR22] Eckmann JP, Olifson Kamphorst S, Ruelle D (1987). Recurrence plots of dynamical systems. Europhys Let.

[CR23] Ellington M (2018). The case for Divisia monetary statistics: a Bayesian time-varying approach. J Econ Dyn Control.

[CR24] Fabretti A, Ausloos M (2005). Recurrence plot and recurrence quantification analysis techniques for detecting a critical regime. Examples from financial market indices. Int J Mod Phys C.

[CR25] Facchini A, Kantz H, Tiezzi E (2005). Recurrence plot analysis of nonstationary data: the understanding of curved patterns. Phys Rev.

[CR26] Facchini A, Mocenni C, Marwan N, Vicino A, Tiezzi E (2007). Nonlinear time series analysis of dissolved oxygen in the Orbetello Lagoon (Italy). Ecol Model.

[CR27] Faggini M (2013). Chaos detection in economic time series: Metric versus topological tools. Adv Manag Appl Econom.

[CR28] Faggini M (2014). Chaotic time series analysis in economics: balance and perspectives. Chaos.

[CR29] Fraser AM, Swinney HL (1986). Independent coordinates for strange attractors from mutual information. Phys Rev A.

[CR30] Hamilton JD (2018). Why you should never use the Hodrick-Prescott filter. Rev Econ Stat.

[CR31] Han L (2019). Correlation predictive modelling of financial markets. Procedia Comput Sci.

[CR32] Hendrickson JR (2014). Redundancy or mismeasurement? A reappraisal of money. Macroecon Dyn.

[CR51] Hodrick RJ, Prescott EC (1980) Postwar U.S. business cycles: an empirical investigation. Discussion Paper No. 451, Carnegie-Mellon University

[CR33] Jadidzadeh A, Serletis A (2019). The demand for assets and optimal monetary aggregation. J Money Credit Bank.

[CR34] Kennel MB, Brown R, Abarbanel HD (1992). Determining embedding dimension for phase-space reconstruction using a geometrical construction. Phys Rev A.

[CR35] Kydland FE, Prescott EC (1990). Business cycles: real facts and a monetary myth. Fed Reserve Bank Minneap Q Rev.

[CR53] Marwan N (2003) Encounters with neighbours. University of Potsdam

[CR54] Marwan N (2006) Command line recurrence plots. http://tocsy.pik-potsdam.de/commandline-rp.php

[CR36] Marwan N (2008). A historical review of recurrence plots. Eur Phys J Spec Top.

[CR37] Marwan N, Webber CL, Webber C, Marwan N (2015). Mathematical and computational foundations of recurrence quantifications. Recurrence quantification analysis: understanding complex systems.

[CR38] Marwan N, Carmen Romano M, Thiel M, Kurths J (2007). Recurrence plots for the analysis of complex systems. J Phys Rep.

[CR39] Marwan N, Schinkel S, Kurths J (2008). Recurrence plots 25 years later. Gaining confidence in dynamical transitions. Europhys Lett.

[CR52] MATLAB Release (2008) The MathWorks, Inc., Natick

[CR40] Packard NH, Crutchfield JP, Farmer JD, Shaw RS (1980). Geometry from a time series. Phys Rev Lett.

[CR41] Ramsey JB, Rothman P (1994). Comment on nonlinear monetary dynamics by DeCoster and Mitchell. J Bus Econ Stat.

[CR42] Ramsey JB, Sayers CL, Rothman P (1988). The statistical properties of dimension calculations using small data sets: Some economic applications. Int Econ Rev.

[CR43] Serletis A (1995). Random walks, breaking trend functions, and the chaotic structure of the velocity of money. J Bus Econ Stat.

[CR44] Serletis A, Andreadis I (2000). Chaotic analysis of US money and velocity measures. Int J Syst Sci.

[CR45] Serletis A, Gogas P (2014). Divisia monetary aggregates, the great ratios, and classical money demand functions. J Money Credit Bank.

[CR46] Serletis A, Shintani M (2006). Chaotic monetary dynamics with confidence. J Macroecon.

[CR500] Serletis A, Xu L (2020) Functional monetary aggregates, monetary policy, and business cycles. J Econ Dynam Control 121:103994

[CR55] Serletis A, Xu L (2021) Consumption, leisure, and money. Macroeconomic Dynamics, (forthcoming)

[CR48] Strozzi F, Zaldivar JM, Zilbut JP (2002). Application of nonlinear time series analysis techniques to high-frequency currency exchange data. Phys A.

[CR56] Takens F (1981) Detecting strange attractors in turbulence. In Rand D, Young LS (eds) Dynamical systems and turbulence, Lecture Notes in Mathematics 898, Springer, Berlin

[CR49] Trulla LL, Giuliani A, Zbilut JP, Webber CL (1996). Recurrence quantification analysis of the logistic equation with transients. Phys Lett A.

[CR59] Xu L, Serletis A (2022). The demand for assets: Evidence form the Markov switching normalized quadratic model. J Money Credit and Banking (forthcoming)

[CR50] Zbilut JP, Webber CL (1992). Embedding and delays as derived from quantification of recurrence plots. Phys Lett A.

